# Identifying and assessing the benefits of interventions for postnatal depression: a systematic review of economic evaluations

**DOI:** 10.1186/s12884-018-1738-9

**Published:** 2018-05-21

**Authors:** Binu Gurung, Louise J. Jackson, Mark Monahan, Ruth Butterworth, Tracy E. Roberts

**Affiliations:** 1 Health Economics Unit, Institute of Applied Health Research, College of Medical and Dental Sciences, University of Birmingham, Edgbaston, Birmingham B15 2TT UK; 2 Cheshire and Mersey Specialist Perinatal Mental Health Service, Thorn Road Clinic, Thorn Road, Runcorn, WA7 5HQ UK

**Keywords:** Postpartum depression, Economic analysis, Parental outcomes, Non-health consequences, QALYs, Outcomes for young children

## Abstract

**Background:**

Economic evaluations of interventions for postnatal depression (PND) are essential to ensure optimal healthcare decision-making. Due to the wide-ranging effects of PND on the mother, baby and whole family, there is a need to include outcomes for all those affected and to include health and non-health outcomes for accurate estimates of cost-effectiveness. This study aimed to identify interventions to prevent or treat PND for which an economic evaluation had been conducted and to evaluate the health and non-health outcomes included.

**Methods:**

A systematic review was conducted applying a comprehensive search strategy across eight electronic databases and other sources. Full or partial economic evaluations of interventions involving preventive strategies (including screening), and any treatments for women with or at-risk of PND, conducted in OECD countries were included. We excluded epidemiological studies and those focussing on costs only. The included studies underwent a quality appraisal to inform the analysis.

**Results:**

Seventeen economic evaluations met the inclusion criteria, the majority focused on psychological /psychosocial interventions. The interventions ranged from additional support from health professionals, peer support, to combined screening and treatment strategies. Maternal health outcomes were measured in all studies; however child health outcomes were included in only four of them. Across studies, the maternal health outcomes included were quality-adjusted-life-years gained, improvement in depressive symptoms, PND cases detected or recovered, whereas the child health outcomes included were cognitive functioning, depression, sleep and temperament. Non-health outcomes such as couples’ relationships and parent-infant interaction were rarely included. Other methodological issues such as limitations in the time horizon and perspective(s) adopted were identified, that were likely to result in imprecise estimates of benefits.

**Conclusions:**

The exclusion of relevant health and non-health outcomes may mean that only a partial assessment of cost-effectiveness is undertaken, leading to sub-optimal resource allocation decisions. Future research should seek ways to expand the evaluative space of economic evaluations and explore approaches to integrate health and non-health outcomes for all individuals affected by this condition. There is a need to ensure that the time horizon adopted in studies is appropriate to allow true estimation of the long-term benefits and costs of PND interventions.

**Electronic supplementary material:**

The online version of this article (10.1186/s12884-018-1738-9) contains supplementary material, which is available to authorized users.

## Background

Postnatal depression (PND), also called postpartum depression, is a non-psychotic, depressive disorder occurring in women within a year after childbirth [1, 2]. It is a common disorder thought to affect 1 in 10 women within the first postpartum year [2]. The period prevalence of minor or major depressive episodes is estimated to be 19.2% in the first three months following childbirth, with 7.1% of mothers experiencing major depressive episodes [3]. Mothers with PND are likely to experience disturbing emotions and feelings common to depression such as anger, guilt, hopelessness, social withdrawal, and those specific to the perinatal period such as sleep deprivation and bonding difficulty [4, 5].

As well as affecting the mother, PND can also affect others within the family. A meta-analysis of studies documenting depression of fathers in the first postpartum year reported that fathers had a 10% risk of experiencing depression and found the correlation between paternal and maternal depression to be positive and moderate [6]. The experience of PND in the parent or parents can potentially lead to marital problems, the withdrawal of social support between parents can compromise adequate care-giving practices of parents, or parent-baby interactions (e.g. the ability of the mother to respond sensitively to her child), that in turn may negatively affect the cognitive, behavioural and social development of the infant in the short and long-term [7–9].

The economic costs associated with PND are significant [10]. In 2002, Petrou and colleagues reported average additional health and social care costs of £392 (2000 prices; UK pound sterling) in women with PND compared to women without PND over the first 18 months post-partum [11]. Recently, Bauer and colleagues estimated that the societal discounted cost of depression during pregnancy and the postnatal period in the long-term was nearly £74,000 per case (2012/13 prices; UK pound sterling) [12]. Around 70% of the projected costs related to the impacts on children, calculated in terms of pre-term birth, mortality, emotional problems, education and conduct, over a period ranging between birth and overall lifetime.

PND has long been considered a major public health problem [13, 14] and a range of PND interventions have been developed in order to prevent or treat the condition. Compared to evaluations of clinical effectiveness of PND interventions, evaluation of their cost-effectiveness within an economic evaluation (comparative analysis of alternative interventions or programmes in terms of both costs and consequences) has been relatively limited [15–17]. The economic evaluation of a PND intervention is essential to understand the value of the intervention relative to other interventions to allow appropriate allocation of healthcare resources [18]. A key consideration in an economic evaluation of an intervention in health conditions like PND, where the impact could potentially go beyond mothers to children, fathers, and could include non-health aspects such as the child’s educational and emotional well-being, is ensuring that all relevant outcomes for all those affected by the intervention are identified and included [19]. Furthermore, it may be necessary to include outcomes that are broader than typical direct health outcomes, as is often the case for public health interventions [20, 21].

Therefore this study was conducted with the aim to systematically review published and unpublished studies of interventions to treat or prevent PND, in which an economic evaluation has been conducted in OECD (Organisation for Economic Cooperation and Development) countries, in order to investigate the outcomes considered and measured. The specific objectives were i) to identify studies of interventions to prevent or treat PND which included an economic evaluation; ii) to ascertain which outcomes were included and how these were measured and valued; and iii) to identify any methodological issues associated with including and measuring outcomes in economic evaluations of PND interventions.

## Methods

A systematic review was conducted in adherence with guidance on methods from the Centre for Review and Dissemination [22] and on reporting from Preferred Reporting of Items for Systematic Review and Meta-Analysis (PRISMA) [23].

### Search strategy

A comprehensive, systematic search strategy was developed through consultation with an information specialist (Additional file 1). The searches were run from database inception to July 2015 in eight healthcare databases: MEDLINE, MEDLINE in-process and other non-indexed citations, EMBASE, PsychINFO, Cumulative Index to Nursing & Allied Health Literature (CINAHL), National Health Service Economic evaluation database (NHS EED), Health Technology Assessment (HTA) and Web of Science (WOS) core collections. The search was not restricted by the publication date or language. Alongside this process, key journals were hand-searched (these were those which appeared most frequently in results of the searches for relevant papers) and reference lists of all the included studies were screened. Furthermore, key researches in the field and members of the *Birmingham Perinatal and Infant Mental Health Forum* were contacted to identify potential published or unpublished literature.

### Inclusion and exclusion criteria

The studies were assessed and selected using the PICOS framework [22] as a guide. The inclusion criteria were: women with or ‘at risk’ of postnatal depression (i.e. those who are pregnant or have given birth within the 12 months), living in OECD countries, and interventions involving preventive strategies (including screening), and any treatments or other interventions for PND. We restricted our focus to OECD countries, in order to compare economic evaluations concerned with similar health care systems. The comparators included placebo, no intervention and current or standard care. In terms of study design, studies that involved a full or partial economic evaluation or that included economic data were potentially eligible for inclusion.

Broadly speaking, the different forms of economic evaluation can be differentiated by how outcomes are considered (although there are also other key differences, for example, in terms of their theoretical foundations) [10]. A cost-utility analysis (CUA) involves consideration of outcomes in terms of quality-adjusted-life-years (QALYs) which combine measurement of quantity and quality of life [18]. In cost-effectiveness analysis (CEA) outcomes are expressed in natural units (e.g. cases detected) and in cost-benefit analysis (CBA) outcomes are valued in monetary terms. Partial forms of economic evaluation include cost-consequence analysis (CCA) where costs and outcomes are presented in a disaggregated form and cost-minimisation analysis (CMA) that is only recommended in certain circumstances where treatments are proven to have identical outcomes [10].

The following were excluded from the review: epidemiological studies reporting incidence/prevalence; costing studies describing costs only; clinical studies describing and evaluating efficacy or effectiveness only; ongoing or incomplete economic evaluations; discussion papers, letters or commentaries.

### Study categorisation

**Fig. 1:**
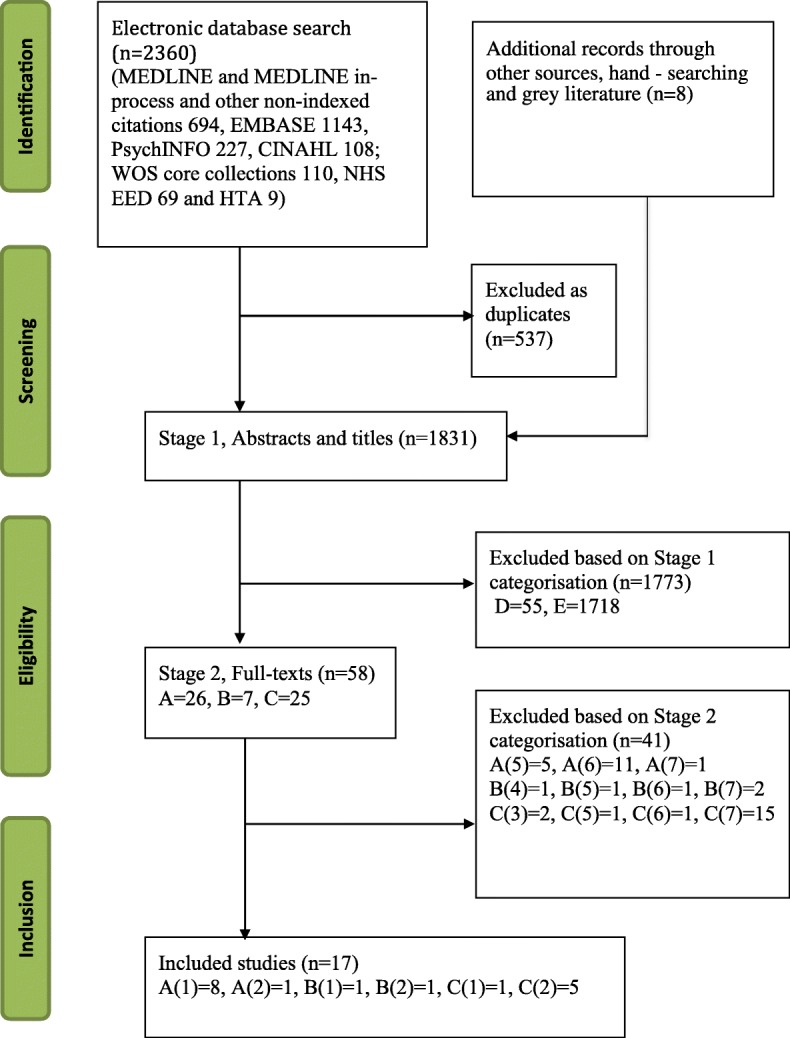
PRISMA flowchart showing the study selection process. Notes: Coding- Stage 1) A. The study involves a formal economic evaluation of PND interventions based on primary and/or secondary data (e.g. previously published studies or other sources); B. The study discusses economic aspects of PND interventions and contains relevant primary and/or secondary data; C. Unclear if the study falls under (A) or (B) but contains useful information; D. The study discusses economic aspects of PND interventions, but is neither (A) nor (B); E. The study is not relevant to the economic evaluation of PND interventions. Stage 2) 1. Full economic evaluation; 2. Partial economic evaluation; 3. Study that measured/valued outcomes of PND interventions but did not consider cost or cost-effectiveness; 4. Other, such as study estimating resource use and/or economic burden of PND and interventions; 5. Secondary study discussing methods or results of economic evaluation; 6. Incomplete economic evaluation of PND interventions (e.g. ongoing studies); 7. Not relevant to the economic evaluation of PND interventions. (See Additional file 2)

A two-stage process outlined by Roberts and colleagues [24] was used to select and categorise studies based on their eligibility and codes for Stage 1 and 2 are provided in Additional file 2 (and as a Footnote for Fig. 1). Stage 1 involved the initial categorisation of studies into categories A to E based on titles and abstracts according to whether the study involved an economic evaluation or included economic data. One author (BG) carried out the initial coding of the studies and another author (LJ) checked the coding. Studies categorised as potentially relevant to the systematic review were carried forward to Stage 2 and assessed for inclusion based on their full-text. One author (BG) carried out the stage 2 coding from 1 to 7, which was then checked by two authors (LJ and MM) and two authors (BG, LJ) assessed the full-texts of all the studies carried forward to Stage 2.

### Data extraction and quality assessment

A data extraction form was used to collect data on the background of the study, details of the outcomes included and any methodological limitations acknowledged by study authors (Additional file 3). The included studies were assessed for quality using an adapted version of Drummond et al.’s checklist [25], which is judged to be suitable for assessing economic evaluations [22]. According to the objectives of the review, the modifications to the checklist included greater focus on outcomes than costs, the addition of a question on the perspective of analysis and the removal of a general question on the study results. The assessment aimed to inform the main analysis rather than to exclude studies based on quality (the full results are available in Additional file 4).

## Results

The electronic search of the databases yielded 2360 studies. A further eight studies were identified as potentially relevant to the review from other sources (key researchers in this field and the forum members). After removing 537 duplicates, 1831 records were categorised based on title and abstract (Stage 1). The full-texts of 58 papers were assessed (Stage 2) and 17 studies were identified for synthesis. Figure 1 presents a PRISMA flow diagram of the articles screened, included or excluded in each stage.

### Study characteristics

The study characteristics are provided in Table 1. The studies were predominantly from the UK (*n* = 13) with a further four studies conducted in Australia [26], Canada [27], New Zealand [28] and the USA [29].

**Table 1:** Study characteristics and aims

Lead author (Year)	Intervention	Comparator	Country	Sample size, N; patient population	Primary aims of economic evaluation	Analytical approach
RCT or cohort-based economic evaluation						
Boath (2003) [39]	PBDU customised treatment (consisting of one or more of the following: individual, couple and family counselling, group therapy, creative therapy, hobbies and activities, stress management, assertiveness training, yoga and relaxation, a group for parents and older children and pharmacotherapy)	RPC	UK	*N* = 60; Women with a baby aged 6 weeks-1 year, with the EPDS score above 12 and with a diagnosis of a major or minor depressive disorder.	Assess the cost-effectiveness of two alternative approaches to the PND treatment.	CEA^a^
Dukhovny (2013) [27]	Telephone-based peer support intervention, access to standard postpartum care	Usual care	Canada	*N* = 610; High-risk women with EPDS score > 9, able to speak English, following a live birth and discharge home	Determine the cost-effectiveness of a peer support intervention to prevent PND.	CEA
Hiscock (2007) [26]	Individual structured maternal and child health consultations, a choice of behavioural interventions, ‘controlled crying’ or ‘camping out’	Usual care	Australia	*N* = 328; Mothers reporting an infant sleep problem in the concurrent 7-month questionnaire	Assess the effectiveness and costs of an intervention targeting infant sleep problems.	CCA
MacArthur (2003) [31]	Redesigned model of community postnatal care (midwifery-led)	Current care	UK	*N* = 36 clusters; All pregnant women registered at the practice antenatally from about 34 weeks gestation.	Develop, implement and test the cost-effectiveness of redesigned postnatal care compared with current care on women’s physical and psychological health.	CCA
Morrell (2000) [32]	Community midwifery support worker	Postnatal midwifery care	UK	*N* = 471; All women who delivered a baby at the recruiting hospital living within the study area, were aged 17 or over, and could understand English.	Measure the effect and the total cost per woman of providing postnatal support at home.	CMA
Morrell (2009) [37]	Health visitor trained to identify and deliver CBA or person-centred approach	Health visitor usual care	UK	*N* = 418; Women at risk of PND indicated by EPDS score ≥ 12, registered with participating GP practices, 36 weeks pregnant during recruitment, who had a live baby and were on a collaborating Health Visitor’s caseload.	Investigate outcomes for postnatal women attributed to the intervention, and to establish its cost-effectiveness.	CUA^a^
Petrou (2006) [33]	Counselling and support package by trained health visitors	RPC	UK	*N* = 151; Women attending clinics at 26–28 weeks of gestation, identified as at high risk of developing PND	Assess the cost-effectiveness of a preventive counselling and support package for women at high risk of developing PND.	CEA
Price (2015) [29]	Enhanced engagement in home visiting via motivational interviewing and brief intervention (CBT and Interpersonal Therapy)	Usual care	USA	*N* = 25; Pregnant and postpartum women in low-income and ethnic minorities communities meeting risk criteria for major depression	Examine the feasibility of enhanced engagement in routine community care over usual care maternal and child health home visiting.	CCA^a^
Sembi (2016) [41]	Telephone peer support	Standard care	UK	*N* = 28; Women > 16 years when giving birth, experiencing depressive symptom indicated by EPDS threshold ≥10 and/or clinical judgement, and potentially receptive to receiving intervention.	Pilot a telephone peer-support intervention for women experiencing PND.	CCA^a^
Wiggins (2004) [35]	Health visitor support or Community group support	Standard services	UK	*N* = 498; Women living in deprived areas, who gave birth in the specified time period and of any ethnicity.	Measure the impact and cost-effectiveness of two alternative strategies for providing support to mothers in disadvantaged inner city area.	CCA^a^
Model-based economic evaluation						
Battye (2012) [30]	Befriending service (telephone helpline and one-to-one support by trained ‘befriender’ volunteers)	No intervention	UK	Quantitative study: *N* = 39 Qualitative study: N not provided	Demonstrate value for money of Acacia Family Support’s service.	CBA
Bauer (2011) [42]	Universal health visiting (postnatal screening using EPDS and treatment [CBT + antidepressant])	Routine postnatal care	UK	Identification: Hypothetical cohort of women for screening. Treatment: Hypothetical cohort of women with moderate to severe PND for the treatment	Identify and analyse the costs and economic pay-offs of PND interventions.	CUA^a^
Campbell (2008) [28]	Routine screening programme (using PHQ-2) and treatments (antidepressants, psychological therapies or social support) according to severity of PND.	Current practice	New Zealand	N not provided; Mothers who gave birth in any 12 month period, regardless of the number of previous births	Evaluate value for money of implementing a screening programme for PND.	CUA^a^, CEA^a^
Hewitt (2009) [36]	Identification 1. EPDS 2. Beck Depression Inventory Treatments 1. Structured psychological therapy 2. Listening visit (Both with preceding additional care)	Current practice	UK	Identification: Hypothetical population of postnatal women (depressed or not) managed in primary care six weeks postnatally Treatment: Hypothetical cohorts of 1000 women with depression in the postnatal period	Identification: Evaluate the cost-effectiveness for a range of feasible identification strategies for PND in primary care. Treatment: Clarify from the NICE guidance whether treatment strategies were cost-effective compared with usual care.	CUA
NCCMH (2014) [38]	Identification 1. EPDS only 2. Whooley questions followed by EPDS 3. Whooley questions followed by PHQ-9 Treatment 1. Facilitated self-help based on CBT principles 2. Listening visits (Both in addition to standard postnatal care)	Standard care	UK	Identification: Hypothetical cohorts of 1000 postnatal women undergoing screening for depression. Treatment: Hypothetical cohorts of 1000 women with sub-threshold/mild to moderate depression	Identification: Assess the relative cost-effectiveness of formal identification methods for PND. Treatment: Assess the cost-effectiveness of different types of psychological and psychosocial, relative to standard postnatal care alone.	CUA^a^, CEA^a^
Stevenson (2010) [40]	Group CBT	RPC	UK	Secondary RCT *N* = 45; Women meeting a standardised PND diagnosis or scoring EPDS threshold ≥12.	Evaluate the clinical effectiveness and cost-effectiveness of group CBT compared with currently used packages.	CUA^a^
Taylor (2014) [34]	Social support (e.g. advocacy, befriending)	No intervention	UK	Estimated *N* = 100; Women assessed as vulnerable to PND, either as a self-referral, or referred via the mid-wife/GP.	Determine the benefits and costs of the Perinatal Support Project to prevent PND.	CBA^a^

*CBA* Cost-Benefit Analysis, *CBA* Cognitive Behavioural Approach, *CBT* Cognitive Behavioural Therapy, *CCA* Cost-Consequence Analysis, *CEA* Cost-Effectiveness Analysis, *CMA* Cost-Minimisation Analysis, *CUA* Cost-Utility Analysis, *EPDS* Edinburgh Postnatal Depression Scale, *NCCMH* National Collaborating Centre for Mental Health, *PBDU* Parent baby day unit, *PHQ* Patient Health Questionnaire, *RCT* Randomised Controlled Trial, *RPC* Routine primary care

^a^Not explicitly stated by authors

A variety of interventions were evaluated in the identified studies that conducted an economic evaluation. Nine studies examined interventions concerned with preventive strategies only [26, 27, 29–35], five studies included both screening and treatment [28, 30, 36–38], and the remaining three studies assessed treatment only [39–41]. The most common type of intervention was modified or enhanced support or care in the perinatal period either from a health professional or via peer support [27, 28, 30–35, 41]. Many of the interventions included cognitive behavioural therapy (CBT), either alone [40], or in combination with other therapies such as antidepressants [42], interpersonal therapy [29], listening visits [37, 38] and a range of customised treatments [39]. A pharmacological intervention was delivered as one of the interventions in three studies only [35, 39, 42]. Various screening tools (Edinburgh Postnatal Depression Scale - EPDS, Whooley questions, Beck Depression Inventory, PHQ-9) were assessed as part of a wider strategy to identify and treat those experiencing PND [28, 36, 38].The comparator adopted was usual, standard or current care/practice/services in nearly all studies. Two studies used ‘no intervention’ as the comparator [30, 34]. Seven studies did not provide a comprehensive description of their comparators [26, 31, 32, 35, 40–42].

All studies investigating screening considered women in their postnatal period [28, 36–38, 42]. Other preventive strategies focused on pregnant [31, 33] and/or postpartum women [26–28, 30, 33, 35]. For treatment interventions, the targeted population were postpartum women with a PND diagnosis and/or women scoring above the threshold of a screening tool [28, 36, 38–42].

There were some differences in terms of the aims of the interventions evaluated. All but three studies evaluated an intervention that focussed on preventing and/or treating PND alone. These three studies focused on improving other aspects of health and well-being in addition to addressing PND, including women’s physical and general health [31, 32], maternal smoking [35] and child injury [35].

There was a greater number of studies that conducted an economic evaluation alongside a Randomised Controlled Trial (RCT) [26, 27, 31–33, 35, 37, 41] or a cohort study [29, 39] compared to studies that undertook decision modelling [28, 30, 34, 36, 38, 40, 42]. Different approaches to analysis were adopted. CUA was the main approach adopted by six of the 17 studies [28, 36–38, 40, 42]. Of these six studies, two further conducted a CEA [28, 38]. Five of the 17 studies adopted a CCA [26, 29, 31, 35, 41] and three studies conducted a CEA only [27, 33, 39]. Two studies carried out a CBA [30, 34] and only one study conducted a CMA [32].

### Health and non-health outcomes

Different types of both health and non-health outcomes were included in the economic evaluations (Tables 2 and 3). All economic evaluations included maternal health outcomes. Thirteen studies included condition-specific outcomes including PND duration [33]; cases recovered or improved [28, 38, 39], cases detected or averted [27, 28]; improvement in PND symptoms [26, 29, 34, 41] or scores from a screening tool [31, 32, 35]. Five studies also used generic outcomes such as well-being [26, 30, 34] and general health [35, 41]. Six studies measured health in terms of QALYs from the maternal perspective [28, 36–38, 40, 42]. However, health outcomes relating to children were considered in only four studies, this was in terms of cognitive functioning [30], sleep [26], temperament [26, 41], and depression [34]. Seven studies acknowledged that outcomes for children and/or partner health were important or likely to be affected but did not include them in their analysis [28, 36–40, 42]. Only three of the seven studies explained their omission; this was typically due to a lack of reliable data [28, 36], or due to missing data [37]. There was no mention of child or partner/family health outcomes in four studies [27, 29, 32, 33].

**Table 2:** Description of outcomes used in RCT or cohort-based economic evaluations

Study (Year)	Intervention	Outcomes	Outcomes other than maternal and health outcomes	How was the outcome measured and/or valued?	Source	Other outcomes measured in the trial but not used/maybe relevant in the economic evaluation	Outcomes acknowledged but excluded
Boath (2003) [39]	PBDU customised treatment	• PND cases recovered	–	CIS	Cohort study	• Anxiety • Work leisure and family life • Marriage quality and other similar dyads	Child and non-health; reasons for their non-inclusion not provided
Dukhovny (2013) [27]	Telephone-based peer support intervention, access to standard postpartum care	• PND cases averted	–	EPDS (threshold of ≤12 for low risk), SCID	RCT	• Anxiety • Loneliness • Satisfaction with intervention	–
Hiscock (2007) [26]	Individual structured maternal and child health consultations, a choice of behavioural interventions, ‘controlled crying’ or ‘camping out’	• Depression symptoms • Mental and physical health scores • Sleep quality and quantity • Infant’s sleep problem (primary outcome) • Infant’s temperament	Child	EPDS (threshold > 9 for PND), SF-12, sleep questions, night waking indicator, Global Infant Temperament Scale	RCT	NA	–
MacArthur^a^ (2003) [31]	Redesigned model of community postnatal care (midwifery-led)	• PND score	–	EPDS (score of ≥13 indicated risk)	RCT	• Physical and Mental Health • Reported morbidity • ‘Good practice’ • Women and professionals views about care	Child; reasons for its non-inclusion not discussed
Morrell^a^ (2000) [32]	Community midwifery support workers	• PND score	–	EPDS (score of ≥12 indicated risk)	RCT	• General health perception • Functional Social Support • Breastfeeding	–
Morrell (2009) [37]	Health visitor trained to identify and deliver CBA or person-centred approach (listening visits)	• QALY	–	The SF-6D, from a subset of SF-36 questions, was calculated. SF-6D scores estimated using UK tariffs.	RCT	• Proportion of at-risk women (primary) • EPDS score • Physical and mental health • Clinical Outcomes in Routine Evaluation • Anxiety • Perceived stressful impact of having a young child • Couples relationship • Cognitive, social and emotional development of infants • Risk of developing autism	Child, partner/family; these outcomes could not included due to missing data
Petrou (2006) [33]	Counselling and support package by trained health visitors	• Duration of PND experienced	–	SCID-II	RCT	Unclear what other outcomes were measured in the trial	–
Price (2015) [29]	Enhanced engagement in home visiting via motivational interviewing and brief intervention (CBT and Interpersonal Therapy)	• Depressive symptoms • Social support	Non-health	Patient Health Questionnaire-9, Sarason’s Social Support Questionnaire-Revised	Cohort study	NA	–
Sembi (2016) [41]	Telephone peer support	• Depressive symptoms • Parent-infant interaction • Anxiety • Emotional support • Parents’ satisfaction and efficacy in their parenting role • Optimistic self-beliefs • Couples relationship • Infant temperament • Perceptions of interventions • General health	Child, non-health	EPDS (score of > 9 or 10 indicated mild depression), CARE-Index, Hospital Anxiety and Depression Scale, Emotional Support Questionnaire, Parenting Sense of Competence scale, Generalised Self-efficacy Questionnaire, Dyadic Adjustment Scale, Infant Temperament Questionnaire and Peer Support Evaluation Inventory, SF-12	RCT	NA	–
Wiggins^a^ (2004) [35]	Health visitor support or Community group support	• PND score	–	EPDS (score ≥ 12 indicated high risk)	RCT	• Child injury • Maternal smoking • Social support • Maternal and child health • Infant feeding • Mother-child interaction • Household resources	–

*CBA* Cognitive Behavioural Approach, *CBT* Cognitive Behavioural Therapy, *ClS* Clinical interview schedule, *EPDS* Edinburgh Postnatal Depression Scale, *PBDU* Parent and baby day unit, *RCT* Randomised Controlled Trial, *SCID* Structured Clinical Interview for Depression, *SF* Short Form, *NA* Not applicable

^a^Study focused on PND and other aspects – other outcomes used in the trial may not necessarily relate to PND

**Table 3:** Description of outcomes used in model-based economic evaluations

Study (Year)	Intervention	Outcomes	Outcomes other than maternal and health outcomes	How was the outcome measured and/or valued?	Source	Key assumptions	Outcomes acknowledged but excluded
Battye (2012) [30]	Befriending service (telephone helpline and one-to-one support by trained ‘befriender’ volunteers)	Short-term • Improvements in mental health • Increased awareness of PND and PND support • Increased coping ability Long-term • Reduced infants behavioural problems • Improved infants cognitive functioning • Family functioning improvement Healthcare professionals and volunteers outcomes also measured.	Child, others^a^ and non-health	Short Warwick-Edinburgh Mental Wellbeing Scale, qualitative interviews and evaluation form	Questionnaires, qualitative interviews, monitoring data, and published studies	Intervention benefits will sustain in the future with only 20% drop-off.	–
Bauer (2011) [42]	Universal health visiting (postnatal screening using EPDS and treatment [CBT+ antidepressant])	• QALY	–	Utilities for depression states derived from secondary sources.	Bennett et al. [51] and Revicki and Wood [52]	Without treatment, PND will sustain with a short-term resolution. Symptoms of moderate-to-severe PND are comparable to those of moderate-to-severe depression.	Child and non-health; reasons for their non-inclusion not provided
Campbell (2008) [28]	Routine screening programme (using PHQ-2) and treatments (antidepressants, psychological therapies or social support) according to severity of PND.	• PND cases detected • PND cases resolved • QALY	–	PHQ-2, Preference weights for QALYs derived from a secondary source.	Secondary sources, Revicki and Wood [52]	Normal utility six-weeks post-treatment in the treatment responders. Non-responders with mild/moderate depression recover within six months of its onset. PND will sustain in undetected cases and non-responders with severe depression. A linear deterioration or improvement between health states over time.	Child and non-health; child outcomes could not be included due to lack of reliable data
Hewitt (2009) [36]	Identification 1.EPDS 2. Beck Depression Inventory Treatments 1. Structured psychological therapy 2. Listening visit (Both with preceding additional care)	• QALY	–	Utility weights derived for QALYs from a secondary source.	Effectiveness estimate from a systematic review and meta-analysis, utility values from Revicki and Wood [52]	Non-responders to treatment and usual care would remain depressed until the model endpoint. Women enter the relevant treatment at 6 weeks postnatally. A linear deterioration or improvement between health states over time.	Child and partner/family; these outcomes could not be included due to lack of reliable data
NCCMH (2014) [38]	Identification 1. EPDS only 2. Whooley questions followed by EPDS 3. Whooley questions followed by PHQ-9 Treatment 1. Facilitated self-help based on CBT principles 2. Listening visits (Both in addition to standard postnatal care)	Identification • QALY Treatment • QALY • PND cases improved and not relapsed	–	EPDS, Whooley question, PHQ-9. Utility weights derived for QALYs from a secondary source.	Effectiveness estimate from meta-analyses, utility values from Sapin and colleagues [53], experts opinion	Identification False negative women could have spontaneous recovery or be identified in the GP follow-up and offered treatment. Only first-line treatments considered and relapse not modelled. Treatment Women who improve remain in the state or relapse until the model endpoint. A linear deterioration or improvement between health states over time.	Child, partner/family and non-health; reasons for excluding non-health outcomes was the lack of relevant evidence
Stevenson (2010) [40]	Group CBT	• QALY	–	Changes in EPDS scores were translated to changes in utility using secondary data.	Data from Morrell et al. [37]	Benefits would sustain over the 6-month period with linear decline afterwards to zero, a year after the treatment.	Child and partner/family; reasons for their non-inclusion not provided
Taylor (2014) [34]	Social support (e.g. advocacy, befriending)	• Increased well-being • Increased chances of employment and higher earnings • Long-term beneficial children outcomes • Reduced use of health and social care services • Increased tax revenues • Volunteers benefits	Child, others^a^ and non-health	Hospital Anxiety and Depression Scale, analysis of a cohort study	Experts, a range of secondary sources	Benefits were estimated from an observational study and an RCT of similar service. Benefits for women and society inferred from experts and a range of published studies.	–

*CBT* Cognitive Behavioural Therapy, *EPDS* Edinburgh Postnatal Depression Scale, *NCCMH* National Collaborating Centre for Mental Health, *PHQ* Patient Health Questionnaire, *QALY* Quality-adjusted-life-year, *RCT* Randomised Controlled Trial

^a^Others include partner/family, volunteers or healthcare professionals

Similarly, non-health outcomes were explicitly considered by four studies only in relation to PND [29, 30, 34, 41]. Nearly all of these studies included outcomes relating to social or emotional support for PND [29, 30, 41]. Other non-health outcomes included mother’s employment and earnings, parent-infant interaction, children’s educational attainment and behavioural problems, couples’ relationships, satisfaction and efficacy in parenting role, and family functioning. Another four studies explicitly acknowledged the significance of non-health effects, but did not include them [26, 28, 38, 39]. A lack of relevant evidence was the main reason stated for excluding potential non-health effects [30, 34, 38].

### Outcome measurement and valuation

Different instruments were used to assess the presence, risk or duration of PND as an outcome in the economic evaluation (Tables 2 and 3). Seven studies employed the Edinburgh Postnatal Depression Scale (EPDS) [26, 27, 31, 32, 35, 38, 41]. However, although the same tool was used, a range of thresholds were used. For example, the thresholds used to define the risk of PND ranged from 9 above [26] to 13 or above [31]. Some defined the EPDS threshold in relation to its level of specificity and sensitivity, by validating it against an existing diagnostic tool [31] or by piloting it on trial participants [32], while others referred to different published sources [26, 27]. Other instruments were also used, with three studies employing the Patient Health Questionnaire [28, 29, 38], two using the Structured Clinical Interview for Depression [27, 33] and one study adopting the Clinical Interview Schedule [39].

In all the economic models that used QALYs, the utility weights were derived from secondary sources (Table 3). Three studies used the same source for utility values [28, 36, 42]. In most cases, the utility values used were based on the health states associated with depressed or general populations rather than women with PND. For other studies involving a monetary valuation of outcomes, some of the valuations of outcomes were based on authors’ own estimates, due to a lack of available data [30].

### Other methodological considerations

#### Study perspective

The most common perspective adopted by the economic evaluations was a National Health Service/Personal Social Services (NHS/PSS) or a healthcare perspective only (*n* = 10) [26, 28, 31–33, 36–38, 40, 41]. Three studies adopted a societal perspective only [34, 39, 42]. Two studies took a societal perspective alongside other perspectives such as a public sector perspective [30], a third-party payer perspective, a healthcare perspective and family perspective [27]. In one study [35], a patient perspective was taken alongside a healthcare perspective.

#### Time horizon and discounting

There were some variations in terms of the time horizon adopted for costs and consequences by the economic evaluations (Table 4). The most common time horizon for outcomes was a year (*n* = 9), followed by six months (*n* = 3), 18 months (*n* = 2), and 12 weeks (*n* = 2). Only two studies adopted time horizons longer than a year, justifying them as necessary due to the short and longer-term impacts of PND [29, 33]. Justifications for adopting a limited time horizon included constraints associated with the trial follow-up period [27, 33], practical limitations and budget constraints [39]. As most studies had the time horizon of a year or less, discounting of benefits was not required. The two studies adopting a longer time period discounted the benefits appropriately at the recommended rate [30, 34].

**Table 4:** Methodological considerations and cost-effectiveness results

Lead author (Year)	Intervention	Perspective (reasons)	Time horizon used in economic evaluation (reasons)	Discounting	Key cost-effectiveness results
RCT or cohort-based economic evaluations					
Boath (2003) [39]	PBDU customised treatment	Societal	6 months (practical considerations, budgetary constraints)	Costs: 6%	“The current treatment of postnatal depression is dominated on the grounds of cost-effectiveness by PBDU treatment. The move from RPC to PBDU would incur an additional cost expended per successfully treated woman of £1945.”
Dukhovny (2013) [27]	Telephone-based peer support intervention, access to standard postpartum care	Societal (US and Canadian guidelines) Third-party payer, Healthcare, Family perspective	12 weeks (RCT time horizon)	No**	The intervention was found to be cost-effective. ICER: CAD $10,009 per case of PND averted There is 95% probability that the program would cost less than CAD $20,196 per PND case averted
Hiscock (2007) [26]	Individual structured maternal and child health consultations, a choice of behavioural interventions, ‘controlled crying’ or ‘camping out’	NHS/PSS*	10, 12 months	No	Benefits Infant sleep problems At 10 months, 56% of intervention and 68% of control mothers reported infant sleep problems (OR 0.61, *p* = 0.04); At 12 months, this fell to 39% vs 55% (OR 0.53, *p* = 0.007). EPDS scores Intervention mothers had lower mean EPDS scores than controls at 12 months (5.9 vs 7.2, *p* = 0.001) and higher mental health (SF-12) scores at both 10 months (48.1 vs 45.0, *p* = 0.001) and 12 months (49.7 vs 46.1, *p* = 0.001). Costs Intervention: £96.93 (SD, £249.37) Control: £116.79 (SD, £ 330.31) Mean difference: £19.44 (95% CI £283.70 to £44.81, *p* = 0.55)
MacArthur (2003) [31]	Redesigned model of community postnatal care (midwifery-led)	Healthcare	12 months	No**	“The cost-consequences analysis established that the costs of the intervention and control care were broadly equivalent. The intervention care costing at a maximum £81.90 more per woman to deliver, but possibly representing a saving of £78.30 per woman, depending on assumptions used.”
Morrell (2000) [32]	Community midwifery support worker	Healthcare	6 weeks (Valid use of EPDS enabling comparability with other trials)	Costs: 5%	Given that health outcomes were similar for both groups, the economic analysis is limited to a comparison of costs between the intervention and control groups. Mean total cost to the NHS at 6 weeks (primary analysis) Intervention group: £635 (SD, £326) The control group: £456 (SD, £291) Mean difference: £180 (95% CI, £126, £232, *p* = 0.001).
Morrell (2009) [37]	Health visitor trained to identify and deliver CBA or person-centred approach	NHS/PSS (NICE guidelines)	6 months	No**	The intervention dominated the comparator for at-risk women at 6 months (primary analysis). However, a significant difference was not observed in the number of QALYs gained in the intervention groups compared to the control group and there was uncertainty associated with the cost and QALY pairs. The probability of CBT being cost-effective was just over 70%***
Petrou (2006) [33]	Counselling and support package by trained health visitors	Healthcare	18 months (RCT time period)	Outcomes: 1.5% Costs: 6%	The intervention is cost-effective compared to RPC. ICER £43.1 per month of PND avoided. The probability that the intervention is cost-effective exceeds 70% once decision makers express a willingness to invest £1000 to prevent each month of PND.
Price (2015) [29]	Enhanced engagement in home visiting via motivational interviewing and brief intervention (CBT and Interpersonal Therapy)	Service providers*	12 weeks	No	Benefits “A decrease in depressive symptoms associated with the intervention that approached statistical significance (*p* = 0.0600). Significant increase in perceived social support (*t* = 3.35, *p* = 0.0027).” Mean Costs Usual care: $158.30 per participant Enhanced Engagement: $147.50 per participant
Sembi (2016) [41]	Telephone peer support	Healthcare	6 months	No	Benefits (primary outcomes) No significant differences between-subjects and improvement in mother-infant interaction. Costs Mean cost of the combined use of NHS resources For the intervention group: £800.67 (SD, £761.74) For standard care group: £1537.80 (SD, £1936.37). It was not possible to conduct a cost-effectiveness analysis due to the small number of patients.
Wiggins (2004) [35]	Health visitor support or Community group support	Healthcare, Patients	12,18 months	6% (costs)	Benefits There was no clear difference in any of the primary outcomes. Maternal depression: Fewer women in the combined intervention group scored over the depression threshold on the EPDS (−3%) than the control group Mean Costs The Support Health Visitor intervention emerged as a relatively expensive intervention to implement compared with the Community Group Support intervention. Support Health Visitor = £3255 (SD, £2253) Community Group Support: £3231 (SD, £3323) Control group: £2915 (SD, £2349)
Model-based economic evaluations					
Battye (2012) [30]	Befriending service (telephone helpline and one-to-one support by trained ‘befriender’ volunteers)	Societal, public sector (demonstrate value to society and healthcare)	Outcomes 3, 6 and 30 years Costs 1 year	3.5%	The befriending service was cost-beneficial to both society and the state. Societal perspective For every £1 invested, the estimated SROI: ▪ £3 over the short term ▪ £4 over the medium term ▪ £6.50 over the longer term Public sector perspective For every £1 invested, the estimated SROI: ▪ £0.20 over the short term ▪ £0.20 over the medium term ▪ £1.50 over the longer term
Bauer (2011) [42]	Universal health visiting (postnatal screening using EPDS and treatment [CBT + antidepressant])	Societal*	12 months	No	Health visiting intervention provided a positive net benefit. ICER £4500 per QALY gained Net monetary benefits £640 per mother (at WTP threshold of £20,000). By extrapolation, this amounts to around £300 million for England.
Campbell (2008) [28]	Routine screening programme (using PHQ-2) and treatments according to severity of PND.	Healthcare	12 months	No**	The proposed routine screening programme appears to be highly cost-effective compared to the current practice from a government perspective. ICERs • NZ $287 per additional case detected • NZ $400 per additional case resolved • NZ $3461 per additional QALY gained
Hewitt (2009) [36]	Identification 1.EPDS 2. BDI Treatments 1. Structured psychological therapy 2. Listening visit (Both with preceding additional care)	NHS/PSS	12 months	No**	Identification ICER The identification strategies were not cost-effective compared to the current practice. EPDS at a cut point of 16: 3£41,204 per QALY gained. Other cut points and BDI cut point 10 were either dominated or had ICERs higher than that of EPDS cut point 16. At each of the three WTP thresholds considered (£20,000, £30,000 and £40,000), the strategy with the highest individual probability of being cost-effective was routine case detection. Treatment Structured psychological therapy was a cost effective treatment** but listening home visits was not cost-effective compared to the current practice. ICER Structured psychological therapy: £17,481 per QALY gained Listening home visits: £66,275 per QALY gained There was 50% probability that structured psychological therapy would be cost-effective at a WTP threshold of £20,000 per QALY gained.
NCCMH (2014)[38]	Identification 1. EPDS only 2. Whooley questions followed by EPDS 3. Whooley questions followed by PHQ-9 Treatment 1. Facilitated self-help based on CBT principles 2. Listening visits (Both in addition to standard postnatal care)	NHS/PSS (NICE guidelines)	Identification 12 months Treatment 12 months 7 weeks	No**	Identification The ‘Whooley questions’ followed by PHQ-9 was estimated to be the most cost-effective identification strategy, however, well above the NICE threshold**. ICER Whooley questions followed by EPDS versus Whooley questions followed by PHQ-9: £45,593 per QALY gained Treatment Facilitated self-help compared with standard care was overall more effective and more costly. ICER Facilitated self-help: £2269 per additional woman improving and not relapsing at the end of the model, or £13,324 per QALY gained. The probability of facilitated self-help being cost effective is 0.59 to 0.72***.
Stevenson (2010) [40]	Group CBT	NHS/PSS (NICE guidelines)	12 months	No**	The group CBT compared with RPC was not found to be cost-effective.*** ICER CBT: £46,462 per QALY gained (95% CI, £37,008 to £60,728).
Taylor (2014) [34]	Social support (e.g. advocacy, befriending)	Societal* (to determine value to society)	12 months-over a lifetime	3.5% (outcomes)	Estimated average direct financial cost of providing support: £2230 per woman. Estimated benefit Using SF-6D: £591–£887 per woman treated Using EQ-5D: £1302–£1954 per woman treated

*Not explicitly stated by authors

**Reasons provided: due to small time horizon

***At a willingness-to-pay threshold of £20,000-£30,000/QALY gained

*BDI* Beck Depression Inventory, *CAD* Canadian, *CBA* Cognitive Behavioural Approach, *CBT* Cognitive Behavioural Therapy, *EPDS* Edinburgh Postnatal Depression Scale, *EQ-5D* EuroQol-5 dimensions, *ICER* Incremental cost-effectiveness ratio, *NCCMH* National Collaborating Centre for Mental Health, *NHS* National Health Service, *NZ* New Zealand, *OR* Odds ratio, *PBDU* Parent and baby day unit, *PHQ* Patient Health Questionnaire, *PSS* Personal Social Services, *QALY* Quality-adjusted-life-year, *RCT* Randomised Controlled Trial, *RPC* Routine primary care, *SD* Standard deviation, *SF-6D* Short Form- 6 dimensions, *SROI* Social return on investment, *WTP* Willingness-to-pay

#### Intermediate outcomes

Some studies used intermediate outcomes such as cases detected or averted [27, 28]. While such outcomes may be meaningful in the context of PND interventions, they can be of more limited general use for commissioners as they do not allow comparison of cost-effectiveness across programme areas [10].

#### Sensitivity analysis

Almost half of the studies did not explore uncertainty around the estimates of outcomes within a sensitivity analysis. Those studies that performed sensitivity analysis mainly conducted a deterministic sensitivity analysis [28, 30, 32, 39, 40, 42] and a few studies conducted probabilistic sensitivity analysis [27, 38, 40].

### Cost-effectiveness of interventions

Of the 11 studies conducting full economic evaluations, 10 reported that the intervention under investigation appeared to be cost-effective (Table 4). Of those 10 studies, three studies found that a combination of PND screening and treatment was cost-effective [28, 37, 42], a further three studies reported that treatments such as psychological therapy, facilitated self-help and customized treatment were more cost-effective than standard care [36, 38, 39], and four studies found positive results for preventive strategies which involved peer support or counselling and other specific support [27, 30, 33, 34]. Group CBT was not found to be cost-effective compared to standard care in one study [40].

## Discussion

The systematic review identified 17 studies of interventions to prevent and/or treat PND, in which an economic evaluation was conducted. The majority of the studies focused on psychological or psychosocial interventions and none focused on pharmacological interventions only. Overall, 10 of the 11 full economic evaluations reported that an intervention was cost-effective. These involved a variety of interventions ranging from additional support from health professionals, peer support and combined screening and treatment strategies which were usually compared with standard care. The review identified a number of methodological issues relating to how outcomes were included, measured and valued in the economic evaluations; these related to whose outcomes were included, the inclusion of relevant health and non-health outcomes, study perspective and time horizon.

Guidelines emphasise the need to identify all relevant outcomes in an economic evaluation [25, 43]. However, only four studies considered health outcomes associated with children [26, 30, 34, 41]. This raises concerns since numerous studies have shown the adverse impacts of PND on the child’s health and development, and on their interaction with their mothers [20, 44]. The exclusion of children’s outcomes from an economic evaluation may mean that an incomplete assessment of cost-effectiveness has been undertaken. For example, an intervention found to be less cost-effective compared to another intervention on the basis of maternal outcomes only, may well be *more* cost-effective when potential benefits to the infant’s health are included. However, there could be potential barriers to considering infants’ outcomes such as lack of robust data, or an inability to measure outcomes directly for children. A further methodological barrier could be related to concerns about increasing the likelihood of findings of false significance (type I error) due to the inclusion of multiple outcomes in an evaluation. Similarly, health outcomes for the father and wider family are potentially relevant and require consideration [19, 21].

Non-health outcomes are relevant and important in the context of PND [18]. However, presently, there is no accepted method to determine which non-health effects are important and how they should be incorporated in an economic analysis [43]. A range of potential approaches for public health interventions have been outlined that allow for the inclusion of health and non-health outcomes (e.g. cost-consequence analysis, cost-benefit analysis etc.) [21]. The focus of most of the studies was exclusively on health, with only four studies measuring some kind of non-health outcomes. Several authors deemed non-health outcomes to be important but did not include them in their evaluation due to challenges such as a lack of reliable and quantifiable data, missing data, and more than one primary outcome being included in the trial.

Some methodological issues were evident relating to the measurement and valuation of outcomes. A detailed analysis of the properties and limitations of the existing instruments used to capture outcomes is essential to inform appropriate ways to measure those outcomes. For example, the frequently used EPDS tool had various cut-off thresholds, indicating differing approaches to using this tool. Many authors of the included studies also mentioned the lack of reliable data on utilities. For example, Stevenson et al. [40] used regression techniques to estimate utilities (based on data from a different trial) but acknowledged that this introduced further uncertainty in the analysis. Other authors used utility weights based on the health states associated with general depression and not PND. Although PND and general depression share some similar symptoms they differ in certain characteristics such as the experience of childbirth and sleep deprivation [44]. If utility estimates do not directly relate to PND, there is a possibility that the utilities may overestimate or underestimate the intervention effects.

A societal perspective is generally considered the most appropriate perspective for PND interventions due to the wide range of impacts associated with the condition [21, 45, 46]. This is in keeping with guidance relating to the economic evaluation of public health interventions, where a perspective broader than the healthcare perspective may be necessary [21]. This would enable outcomes beyond health to be considered, such as those relating to education, housing, crime etc. However, the results of this review demonstrated that only five studies adopted a societal perspective.

Another recurrent issue observed was the limited time length adopted by most of the studies. The time horizon was no more than 18 months in the trial-based economic evaluations. It can be argued that important differences between the interventions may not be captured using short time-horizons. For example, a prospective longitudinal study showed that the children, who were adversely affected in their infancy due to their mother’s PND at 3 months postpartum, experienced more problems with intellectual and academic performance at 11 years of age compared to the children of healthy mothers [20] and those problems could have potential economic consequences such as additional school support costs and productivity losses from leaving school without qualifications [10]. Thus, studies adopting a longer time horizon are needed to be able to capture the long-term effects of PND.

This review has several strengths. Systematic and rigorous processes were adopted to identify and assess studies. A comprehensive search strategy was implemented which also included searches for unpublished reports. Both prevention and treatment strategies were included, providing a holistic overview of several methodological issues concerning outcome identification and measurement for the economic evaluation of PND interventions. Using established criteria [25] a quality appraisal process was undertaken analysing all key elements relating to outcomes.

Nonetheless, the review is subject to some limitations. Firstly, potential studies may have been missed by the search strategy either due to inadequate classifications of economic terms in the databases or due to the different ways interventions to improve mental health in the postpartum period can be coded depending on the type or the focus of intervention (e.g. on the mother, the infant etc.) [47]. Secondly, since we could not find detailed guidelines focussed on economic evaluations of PND interventions, our analysis of the quality of the studies was based on generic guidance. Lastly, an in-depth analysis of evidence on the clinical and cost-effectiveness of the interventions was beyond the review’s scope.

This is the first systematic review to examine the approaches taken and types of outcomes used in economic evaluations of PND interventions (for prevention and treatment). A systematic review undertaken by the National Collaborating Centre for Mental Health [38] was concerned with accumulating evidence on the cost-effectiveness of interventions to prevent or treat mental health problems in pregnancy and the postnatal period. More recently a systematic review [16] was conducted to inform parameters for a model-based economic evaluation of antenatal and postnatal interventions for pregnant and postnatal women to prevent PND. While these studies attempted to identify economic evaluations of PND interventions, they did not explore the methodological issues associated with the approaches taken and outcomes adopted in the studies.

The findings of this review highlight several implications for future research. Future economic evaluations should identify and consider the full range of potential outcomes that are relevant in the context of PND: health and non-health outcomes, maternal, family and child outcomes. The development of new methods and refinement of existing approaches that can incorporate both health and non-health benefits of intervention are essential for a complete evaluation of the cost-effectiveness of PND interventions. The list of outcomes generated from this review, as a preliminary framework, could be refined further through engagement with key stakeholders including mothers, family members, clinicians and healthcare commissioners to reach consensus on what outcomes are important for use in economic evaluations of PND interventions and in wider evaluations of interventions and services. Given the wide range of impacts associated with PND, in order to allow a full assessment of costs and consequences, a societal perspective should be considered. However, key challenges remain around the monetary valuation of outcomes to enable analyses adopting a societal perspective to be carried out more robustly. Research addressing this issue could explore methods that have been used before in relation to general depression [48, 49]. Similarly, if QALYs are used as outcome, there is an urgent need to address the paucity of estimates of health state utility values relevant to PND. The limited time period inherent in trial-based economic evaluations could be overcome by exploiting modelling techniques that extrapolate outcomes and costs over an extended timeframe [50] and the most appropriate time horizons could be further explored in consultation with decision-makers. In all types of economic evaluation, robust sensitivity analyses will need to be undertaken to explore the implications associated with uncertainty around outcome estimates.

## Conclusion

This systematic review has demonstrated that very few economic evaluations included and identified all outcomes relevant to PND interventions. For example, outcomes for the child were not included in most studies, and only a minority included non-health outcomes. Thus, the review paves the way for further work to explore new approaches and methods that enable inclusion of relevant health and non-health outcomes. In addition, the time horizons adopted in the studies did not allow long-term outcomes for the child to be addressed, which have been shown to be important for PND. The review also shows that a broader perspective can facilitate the assessment of the overall impact of interventions in this area. To achieve optimal policy decisions for interventions to prevent and treat PND, addressing these methodological issues is essential.

## Additional files

Additional file 1: A search strategy carried out in Ovid MEDLINE (from 1946 to July Week 1 2015). (DOCX 18 kb)

Additional file 2: Categorisation Criteria (details of the two-stage process used for study screening and selection). (DOCX 16 kb)

Additional file 3: Description of key methodological issues relating to outcomes (limitations as acknowledged by the authors of the included studies). (DOCX 20 kb)

Additional file 4: Results of study assessment using Drummond’s checklist (adapted). (DOCX 21 kb)

## Abbreviations

CBA: Cost-benefit analysis
CCA: Cost-consequence analysis
CEA: Cost-effectiveness analysis
CINAHL: Cumulative Index to Nursing & Allied Health Literature
CMA: Cost-minimisation analysis
CUA: Cost-utility analysis
EPDS: Edinburgh postnatal depression scale
HTA: Health Technology Assessment
NHS EED: National Health Service Economic evaluation database
NHS: National Health Service
NICE: National Institute of Health and Care Excellence
OECD: Organisation for Economic Co-operation and Development
PND: Postnatal depression
QALY: Quality-adjusted-life-year
RCT: Randomised controlled trial
