# Supplementary material for: Identifying and assessing the benefits of interventions for postnatal depression: a systematic review of economic evaluations
Source: BMC Pregnancy Childbirth. 2018 May 21;18:179. doi: 10.1186/s12884-018-1738-9 (PMC5963067; doi:10.1186/s12884-018-1738-9)
Supplement: Supplementary file 1 — A search strategy carried out in Ovid MEDLINE (from 1946 to July Week 1 2015). (DOCX 18 kb) [file 12884_2018_1738_MOESM1_ESM.docx]

**Additional file 1.** A search strategy carried out in Ovid MEDLINE(R) (from 1946 to July Week 1 2015)

| **ID** | **Search terms** | **Results** |
| --- | --- | --- |
| 1 | exp Pregnancy/ | 742853 |
| 2 | (pregnan* or gestation* or childbearing).ti,ab,kw. | 462431 |
| 3 | exp Pregnancy Complications/ | 355802 |
| 4 | perinatal care.ti,ab,kw. or exp Perinatal Care/ | 8352 |
| 5 | mother-child relation$.ti,ab,kw. or exp Mother-Child Relations/ | 18221 |
| 6 | (postpartum or post-partum or postnatal or post-natal or perinatal or peri-natal or prenatal or pre-natal or antenatal or ante-natal or matern*).ti,ab,kw. | 375962 |
| 7 | exp Depression/ | 83343 |
| 8 | depress$.ti,ab,kw. | 315540 |
| 9 | exp mental disorder/ | 1003171 |
| 10 | exp mental health/ | 23631 |
| 11 | (mental$ adj (health or disorder$ or disease$ or illness$ or problem$)).ti,ab,kw. | 108760 |
| 12 | exp Cost-Benefit Analysis/ | 63578 |
| 13 | (cost$ adj2 (effective$ or utilit$ or benefit$ or consequence$ or minimi$)).ti,ab,kw. | 92701 |
| 14 | economic evaluation$.ti,ab,kw. | 6597 |
| 15 | (decision adj (analy$ or model$ or tree$)).ti,ab,kw. | 10149 |
| 16 | (cost$ or economic$ or pharmacoeconomic$).ti. | 111224 |
| 17 | quality-adjusted life year$.ti,ab,kw. or exp Quality-Adjusted Life Years/ | 10296 |
| 18 | exp "costs and cost analysis"/ or exp Health Care Costs/ | 191444 |
| 19 | exp Economics, Pharmaceutical/ or exp Economics, Medical/ or Economics/ or exp Economics, Hospital/ | 62922 |
| 20 | or/1-6 | 1021498 |
| 21 | or/7-11 | 1248132 |
| 22 | 20 and 21 | 57183 |
| 23 | or/12-19 | 331757 |
| 24 | 22 and 23 | 767 |
| 25 | limit 24 to humans | 694 |
