# Supplementary material for: Identifying and assessing the benefits of interventions for postnatal depression: a systematic review of economic evaluations
Source: BMC Pregnancy Childbirth. 2018 May 21;18:179. doi: 10.1186/s12884-018-1738-9 (PMC5963067; doi:10.1186/s12884-018-1738-9)
Supplement: Supplementary file 2 — Categorisation Criteria (details of the two-stage process used for study screening and selection). (DOCX 16 kb) [file 12884_2018_1738_MOESM2_ESM.docx]

**Additional File 2.** Categorisation Criteria

**Stage 1**

A – The study involves a formal economic evaluation of PND interventions based on primary and/or secondary data (e.g. previously published studies or other sources);

B – The study discusses economic aspects of PND interventions and contains relevant primary and/or secondary data;

C – Unclear if the study falls under (A) or (B) but contains useful information;

D – The study discusses economic aspects of PND interventions, but is neither (A) nor (B);

E – The study is not relevant to the economic evaluation of PND interventions.

**Stage 2**

1. Full economic evaluation;
2. Partial economic evaluation;
3. Study that measured/valued outcomes of PND interventions but did not consider cost or cost-effectiveness;
4. Other, such as study estimating resource use and/or economic burden of PND and interventions;
5. Secondary study discussing methods or results of economic evaluation;
6. Incomplete economic evaluation of PND interventions (e.g. ongoing studies);
7. Not relevant to the economic evaluation of PND interventions
