# Supplementary material for: Identifying and assessing the benefits of interventions for postnatal depression: a systematic review of economic evaluations
Source: BMC Pregnancy Childbirth. 2018 May 21;18:179. doi: 10.1186/s12884-018-1738-9 (PMC5963067; doi:10.1186/s12884-018-1738-9)
Supplement: Supplementary file 3 — Description of key methodological issues relating to outcomes (limitations as acknowledged by the authors of the included studies). (DOCX 20 kb) [file 12884_2018_1738_MOESM3_ESM.docx]

**Additional file 3.** Description of key methodological issues related to outcomes

| **Lead author (Year)** | **Key issues as stated by the authors** |
| --- | --- |
| Battye (2012) | - No common accepted method for identifying financial values for a number of the benefits identified in this analysis e.g. own estimates applied to value improved mental health. - Range of possible benefits excluded due to insufficient available evidence. - The major benefit of improved health falling to service users was excluded by taking the narrow perspective of costs and benefits to the state. |
| Bauer (2011) | - Important to include in any future evaluation the economic costs of negative consequences (e.g. behavioural, emotional) for the children and possibly fathers over a longer time-horizon. |
| Boath (2003) | - The effect of PND on the infant’s development (e.g. emotional, intellectual) and of infant characteristics (e.g. irritability) on the mother was excluded although a societal perspective was taken, introducing a risk of omitting some relevant benefits. - Limited time horizon, uncertain whether the results will alter on using a longer window. |
| Campbell (2008) | - Benefits of a PND screening are not limited to maternal health outcomes, with health benefits accruing to their children and to society. - Lack of reliable and quantifiable data linking maternal PND and its resolution to cases of child maltreatment or child development. |
| Dukhovny (2013) | - EPDS, was used as the primary marker of effectiveness in the trial due to limitations with diagnostic tool such as SCID. - QALYs were not used because of resource limitations. - Limited time horizon. |
| Hewitt (2009) | - Utility estimates were derived from studies of general depressed population due to limited published data. - Potential impact on other family members or the infant was excluded due to a lack of reliable evidence. - Potential insensitivity of the QALY in mental health context should be considered when interpreting the results. |
| Hiscock (2007) | - Non-blinding of nurses and mothers to group membership, and mothers’ self-reported outcomes may introduce bias towards intervention benefits. |
| MacArthur (2003) | - More than one primary outcome measure specified for the trial constrained the economic analysis. - Not able to translate the effectiveness measures into QALYs or other standardised metrics. - Likely there are benefits to children attributable to the mothers’ improved psychological well-being. |
| Morrell (2000) | - A generic measure such as QALYs would have allowed comparison with other NHS programmes. However, the main purpose of the analysis was to compare total costs and benefits for the intervention and control group. - Limited time period. |
| Morrell (2009) | - Utility estimates were produced from two or more individual observations. - Likely that the intervention could have had an impact on the baby, other children and even the partner. - The collected outcome and cost data associated with the baby and partner were dropped as they compounded the missing data problems. |
| NCCMH (2014) | - Utility weights were from the general population with depression under antidepressant medication and not specific to women with PND. - Health outcomes of the babies and the wider family were excluded, as relevant data were unavailable. - Likely the treatment benefits are higher since improvement in mother’s improvement has a significant positive impact on babies’ development and on wider family’s well-being. - QALYs do not capture process characteristic of the intervention. |
| Petrou (2006) | - Preference-based outcome measure such as the QALY would have been more useful for comparative purposes. - Limited horizon is likely to have underestimated the long-term cost-effectiveness of the intervention. |
| Price (2015) | - Possible other factors may influence the change in outcome scores observed between the two groups. |
| Taylor (2014) | - Estimation technique and the transfer of some outcomes into monetary terms hold uncertainty. - Unable to put a monetary value on the longer-term beneficial impacts on the children and other outcomes. - Likely further positive effects were not captured by the analysis (e.g. improvements in mother-child relations). Monetising them is likely to make the benefits significantly greater. |
| Sembi (2016) | - Findings reliability is limited by the significant loss to follow-up and the inability to extract the impact of other interventions. |
| Stevenson (2010) | - Any additional health benefits for partners and children have been excluded. - Uncertainty in utility measurements as the study relied on a regression of EPDS to SF-6D. |
| Wiggins (2004) | - Women’s satisfaction is not valued as an outcome. - Missing EPDS data |
