# Supplementary material for: Identifying and assessing the benefits of interventions for postnatal depression: a systematic review of economic evaluations
Source: BMC Pregnancy Childbirth. 2018 May 21;18:179. doi: 10.1186/s12884-018-1738-9 (PMC5963067; doi:10.1186/s12884-018-1738-9)
Supplement: Supplementary file 4 — Results of study assessment using Drummond’s checklist (adapted). (DOCX 21 kb) [file 12884_2018_1738_MOESM4_ESM.docx]

**Additional file 4.** Result of study assessment using adapted Drummond et al. checklist (Drummond et al., 1996)

| **Lead author (Year)** | **A well-defined question** | **Analysis perspective** | **Description of comparators** | **Source of intervention effectiveness** | **Important outcomes for each alternative** | **Outcome measured in appropriate physical**  **units** | **Outcomes valued credibly, where applicable** | **Discounting of the costs and benefits** | **Incremental analysis of competing costs and benefits** | **Sensitivity analysis undertaken in the estimates of benefits** |
| --- | --- | --- | --- | --- | --- | --- | --- | --- | --- | --- |
| Battye (2012) | Y | Y  (Societal, public sector) | Y | ? (Cohort study, no control group) | Y | Y | Partly (own estimates for some outcomes) | Y (3.5%) | N | Y (DSA) |
| Bauer (2011) | Y | Partly* (Societal-NS) | N | Y (range of secondary sources) | Partly** | Y | ? | NR | Y | Y (DSA) |
| Boath (2003) | Y | Partly* (Societal) | Y | ? (Cohort study) | Partly** | Y | NA | Y (6% for costs) | Y | Y (DSA) |
| Campbell (2008) | Y | Partly* (Healthcare) | Y | Y (range of secondary sources) | Partly** | Y | ? | NR | Y | Y (DSA) |
| Dukhovny (2013) | Y | Y (Societal and other perspectives in sensitivity analysis) | Y | Y (RCT) | Partly** | Y | NA | NR | Y | Y (PSA) |
| Hewitt (2009) | Y | Partly* (NHS/PSS) | Y | Y (A systematic review and meta-analysis) | Partly** | Y | ? | NR | Y | N |
| Hiscock (2007) | Y | Partly* (NHS/PSS-NS) | N | Y (RCT) | Y | Y | NA | NR | N | N |
| MacArthur (2003) | Y | Partly* (NHS) | N | Y (RCT) | Partly** | Y | NA | NR | N | N |
| Morrell (2000) | Y | Partly* (NHS) | N | Y (RCT) | Partly** | Y | NA | Y (5% for costs) | Y (only costs) | N |
| Morrell (2009) | Y | Partly* (NHS/PSS) | Y | Y (RCT) | Partly** | Y | ? (UK tariffs) | NR | Y (not reported but used to produce CEAC) | N |
| NCCMH (2014) | Y | Partly* (NHS/PSS) | Y | Y (guideline meta-analysis) | Partly** | Y | ? | NR | Y | Y (PSA) |
| Petrou (2006) | Y | Partly* (Healthcare) | Y | Y (RCT, not cited) | Partly** | Y | NA | Y (1.5% for outcomes; 6% for costs) | Y | N |
| Price  (2015) | Y | Partly* (Service providers) | Y | ? (Cohort study) | Partly** | Y | NA | NR | N | N |
| Taylor (2014) | Y | Partly* (Societal) | Y | Y (RCT and observational study) | Y | Y | ? (HADS converted to QALYs and then expressed in monetary units) | Y (3.5% for outcomes) | Y | N |
| Sembi (2016) | Y | Partly* (NHS, service providers) | N | Y (Pilot RCT) | Y | Y | NA | NR | N | N |
| Stevenson (2010) | Y | Partly* (NHS/PSS) | N | Y (RCT found from a systematic review) | Partly** | Y | ? | NR | Y | Y (DSA, PSA) |
| Wiggins (2004) | Y | Partly* (NHS/PSS, patients) | N | Y (RCT) | Partly** | Y | NA | Y (6% for costs) | N | N |
| Wiggins (2004) | Y |  | N | Y (RCT) | Partly* | Y | NA | Y (for costs) | N | N |

Note: Y=Yes, N=No, NA=Not Applicable, NR=Not Required (due to short time horizon); CEAC=Cost-effectiveness Acceptability Curve, DA=Deterministic sensitivity analysis, HADS=Hospital Anxiety and Depression Score, NHS=National Health Service, PSA=Probabilistic sensitivity analysis, PSS=Personal Social Services, QALY=Quality-adjusted-life-years, RCT=Randomised Controlled Trial

* Flagged as an issue when healthcare and societal perspective were not undertaken in the main or sensitivity analysis.

** Considered a concern when relevant outcomes did not include the child outcomes.

A question mark in the ‘outcomes valued credibly’ column meant that the utilities were not derived from the PND population and/or from the UK sample.
